# Development and Validation of 13-plex Luminex-Based Assay for Measuring Human Serum Antibodies to *Streptococcus pneumoniae* Capsular Polysaccharides

**DOI:** 10.1128/mSphere.00128-18

**Published:** 2018-08-08

**Authors:** Danka Pavliakova, Peter C. Giardina, Soraya Moghazeh, Shite Sebastian, Maya Koster, Viliam Pavliak, Andrew McKeen, Roger French, Kathrin U. Jansen, Michael Pride

**Affiliations:** a Vaccine Research and Development and Early Clinical Development Biostatistics, Pfizer Inc., Pearl River, New York, USA; b Affinivax, Cambridge, Massachusetts, USA; c Biotechnology Clinical Development Statistics, Pfizer Inc., Pearl River, New York, USA; Parasitology Services

**Keywords:** Luminex, development, multiplex, pneumococcal, validation

## Abstract

A Luminex-based direct immunoassay (dLIA) platform has been developed to replace the standardized pneumococcal enzyme-linked immunosorbent assay platform. The multiplex dLIA simultaneously measures the concentration of serum immunoglobulin G (IgG) antibodies specific for pneumococcal capsular polysaccharide (PnPS) serotypes 1, 3, 4, 5, 6A, 6B, 7F, 9V, 14, 18C, 19A, 19F, and 23F. The assay uses poly-l-lysine (PLL)-conjugated PnPS, chemically coupled to spectrally distinct Luminex microspheres. Assay validation experiments were performed using residual human serum samples obtained from 13-valent pneumococcal conjugate vaccine (13vPnC) clinical studies. Assay results are expressed as IgG antibody concentrations in micrograms per milliliter using the international reference serum, 007sp. The lower limit of quantitation (LLOQ) for all serotypes covered in the 13-plex dLIA fell within the range of 0.002 to 0.038 µg/ml serum IgG. The difference between the lower limit and upper limit of the assay range was >500-fold for all serotypes, and assay variability was <20% relative standard deviation (RSD) for all serotypes. IgG antibody measurements were shown to be serotype-specific (some cross-reactivity was observed only between the structurally related serotypes 6A and 6B as well as 19A and 19F), and no interference was observed between the serotypes when the assay was performed in the 13-plex format compared to the singleplex assays. The 13-plex dLIA platform developed by Pfizer Inc. generates up to 143 test results in a single 96-well plate and is a suitable replacement of the enzyme-linked immunosorbent assay (ELISA) platform for evaluating vaccine clinical trials.

**IMPORTANCE** The pneumococcal enzyme-linked immunosorbent assay (ELISA) measures IgG antibodies in human serum, and it is an important assay that supports licensure of pneumococcal vaccines. The immune correlate of protection, 0.35 µg/ml of IgG antibodies, was determined by the ELISA method. Pfizer has developed a new Luminex-based assay platform to replace the ELISA. These papers describe the important work of (i) validating the Luminex-based assay and (ii) bridging the immune correlate of protection (0.35 µg/ml IgG) to equivalent values reported by the Luminex platform.

## Abstract (precis)

The pneumococcal enzyme-linked immunosorbent assay (ELISA) measures IgG antibodies in human serum, and it is an important assay that supports licensure of pneumococcal vaccines. The immune correlate of protection, 0.35 µg/ml of IgG antibodies, was determined by the ELISA method. Pfizer has developed a new Luminex-based assay platform to replace the ELISA. These papers describe the important work of (i) validating the Luminex-based assay and (ii) bridging the immune correlate of protection (0.35 µg/ml IgG) to equivalent values reported by the Luminex platform.

## INTRODUCTION

Streptococcus pneumoniae, a significant human pathogen, is a leading cause globally of meningitis, bacteremia, pneumonia, and acute otitis media. There are more than 90 distinct *S. pneumoniae* serotypes, although only a portion of these are most commonly associated with severe disease. Licensed vaccines protect infants and adults from disease caused by the most common serotypes (1–3). Protection against pneumococcal infection is mediated by functional serum immunoglobulin G (IgG) antibodies that are specific for pneumococcal capsular polysaccharide (PnPS). Serum IgG measurements are the basis for licensure of PnPS-containing pneumococcal conjugate vaccines in infants (4–7). The World Health Organization’s (WHO) Expert Committee on Biological Standardization endorsed a standardized pneumococcal enzyme-linked immunosorbent assay (ELISA) (http://www.vaccine.uab.edu/) to be used in the evaluation of humoral immune responses to pneumococcal vaccines in infants. In the WHO ELISA (8), each test yields one serotype-specific result per sample on a given assay plate, and adding tests to accommodate additional serotypes requires a significantly greater volume of serum, which is already limited, given the testing requirements of concomitantly administered pediatric vaccines. Microsphere-based Luminex immunoassays overcome this limitation by using spectrally distinct fluorescent microspheres as the solid support matrix onto which the target antigens are bound to simultaneously measure antibodies against multiple analytes from a single reaction well (9–15). For evaluation of humoral immune responses to pneumococcal vaccines, spectrally distinct fluorescent microspheres are coated with different PnPSs, which allows detection of multiple antigen-specific IgG antibodies simultaneously in a single reaction well, thus reducing analysis time and cost.

This paper describes the development and validation of a 13-plex Luminex-based assay to quantify IgG antibodies to capsular PnPS in human serum. The correlation of the 13-plex assay platform to the WHO ELISA platform was studied in detail using sera from infants who were vaccinated with 7vPnC or 13vPnC (16).

## RESULTS

### Optimization of the coupling of PnPS-PLL conjugates to the microspheres.

Bead coupling was optimized using factorial design of experiments (DoEs). The following factors were evaluated: concentrations of antigen (PnPS–poly-l-lysine [PLL]) and EDC/sNHS [1-ethyl-3-(3-dimethylaminopropyl)carbodiimide/sulfo-N-hydroxysulfosuccinimide], activation and coupling buffers, coupling reaction time, microsphere starting concentration, volume of coupling reaction, microsphere lots, and PnPS-PLL conjugate lots.

Two buffers (Dulbecco’s phosphate-buffered saline [DPBS] and HEPES; Thermo Fisher Scientific) were compared for use in bead activation and coupling. The EDC and sNHS concentrations were assessed to achieve maximum assay sensitivity and robustness. Microspheres were coupled to the PnPS-PLL conjugates after activation at various concentrations (100, 1,000, and 5,000 µg/ml) of EDC and sNHS. Microspheres that had been prepared using an EDC/sNHS concentration of 5,000 µg/ml showed the highest and most consistent MFI (median fluorescent intensity) signals in analyzing the reference standard serum. To determine the optimal concentration of PnPS-PLL conjugates for microsphere coating, the microspheres (1.25× 10^7^ per reaction) were coupled with 500 µl of 0.2, 0.5, and 1 µg/ml solutions of a PnPS-PLL conjugate (serotype 1, 3, 4, 5, 6A, 6B, 7F, 9V, 14, 18C, 19A, 19F, or 23F). The PnPS-PLL concentration that achieved acceptable sensitivity while maintaining robust assay performance was 0.5 or 1 µg/ml, depending on the serotype (data not shown). Optimal coupling time was 120 min for all serotypes (data not shown).

### Robustness of 13-plex dLIA.

Assay robustness is the ability of an assay to remain unaffected by small but deliberate variations in the test method. Here, design of experiments (DoEs) was performed to evaluate optimal assay conditions and robustness. Reference standard serum, a panel of 11 positive human sera, and 3 quality control samples (QCS) were analyzed in the DoEs. Results were used to estimate percent relative standard deviation (%RSD) for each factor and the total standard deviations for all factors. The following factors were studied: day, primary and secondary antibody incubation times, and temperature. Both primary and secondary antibody incubation times (60 to 120 min) and primary and secondary incubation temperatures (18 to 25°C) were evaluated in DoEs; the robustness of the concentrations of the secondary antibody (2, 3.3, and 5 µg/ml) was evaluated in discrete experiments. For each serotype, a variance component analysis was carried out to estimate the variability of each factor and selected interactions. Overall, the results of the DoEs demonstrated that the sample concentrations generated from the assays performed within the specified condition ranges were similar; total variability was less than 20% for all of the serotypes except serotype 6A, where the total variability was 21% (Table 1). Assay conditions were defined to be robust within the evaluated ranges, where primary and secondary incubation times were between 60 and 120 minutes, with primary and secondary incubation temperatures of 18 to 25°C and the concentration of secondary antibody at 3.3 µg/ml (data not shown).

**TABLE 1:** Results from assay robustness DoEs^a^

Serotype	RSD (%)						
	Day	Primary Ab time	Primary Ab temp	Secondary Ab time	Secondary Ab temp	Residual	Total
1	0.00	0.00	0.00	0.00	2.80	13.25	13.95
3	1.51	5.52	12.97	0.00	1.61	10.87	19.07
4	0.62	4.40	0.00	0.00	3.07	14.17	15.72
5	0.00	6.42	0.00	1.16	1.54	14.01	16.32
6A	0.52	6.73	11.69	0.00	2.28	13.87	21.01
6B	0.00	0.00	3.29	0.94	2.06	12.13	13.35
7F	0.61	9.29	8.20	1.13	0.86	13.61	19.97
9V	1.95	1.51	0.00	0.29	1.79	11.31	12.28
14	0.00	0.00	0.00	0.00	3.02	12.79	13.65
18C	0.50	0.61	0.00	0.00	1.83	10.75	11.61
19A	0.95	0.04	3.97	0.00	1.34	13.55	18.89
19F	1.29	3.96	0.00	0.00	2.63	12.42	13.80
23F	0.53	0.00	6.31	0.00	2.89	11.59	13.86

a Primary and secondary antibody (Ab) incubation times, 60, 90, and 120 min; primary and secondary Ab incubation temperatures, 18, 20, and 25°C. Factorial design of experiments (DoEs) was performed to evaluate assay robustness. The following factors were studied: day (analyst), primary antibody incubation time and temperature, and secondary antibody incubation time and temperature. Primary and secondary antibody incubation times were evaluated in a range from 60 to 120 minutes and primary and secondary incubation temperature in a range from 18 to 25^o^C. Percent relative standard deviation (%RSD) was estimated for variations in test sera concentrations due to the factors evaluated. For each serotype, a variance component analysis was carried out to estimate the variability of each factor. The individual components of %RSD are not additive, because the effects of each component have a different weight on the total %RSD. The %RSD total is calculated by using the following formula (36): RSD = log*^e^*(1 + %RSD^2^).

### Performance of the reference standard serum.

**FIG 1:**
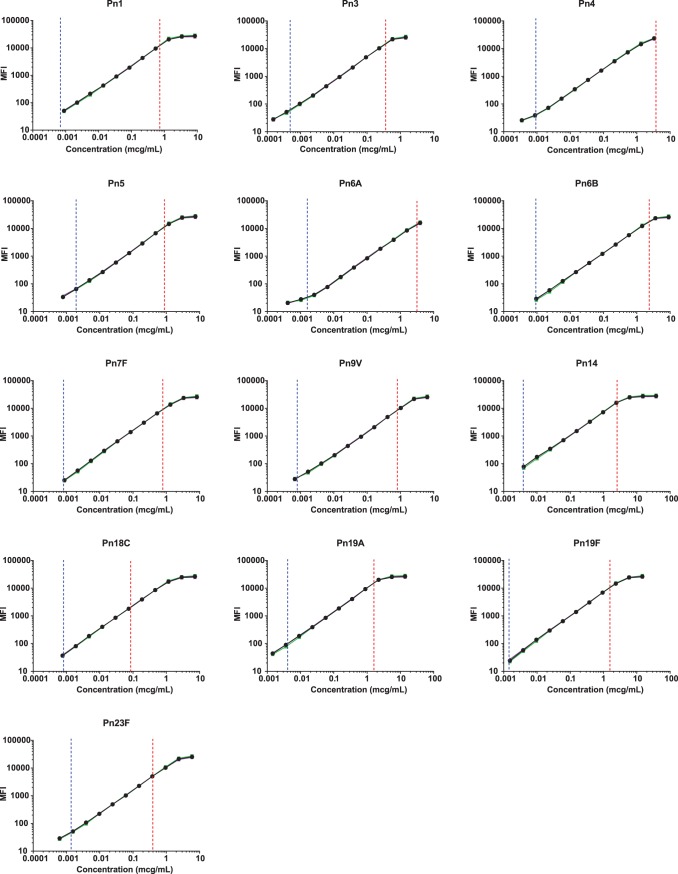
Dynamic ranges of anti-PnPS IgG Reference standard serum 007sp for each of 13 serotypes. MFI signal data for the reference standard curve are on the *y* axis, and specific IgG antibody concentration data are on the *x* axis. The blue line indicates the assay range lower limit as determined in the assay validation; the red line indicates the assay range upper limit.

Reference standard serum 007sp, which has assigned IgG antibody concentrations for all 13 serotypes (17), was used as the reference standard in the 13-plex dLIA. Eleven 2.5-fold dilutions of 007sp serum were performed and used in the assay. A log/log-linear regression model was used to fit the reference standard curve. Figure 1 shows the reference standard serum dilution profiles for each of the 13 PnPS serotypes. The lower (LL) and upper limit (UL) of the assay range, as determined during assay validation (see results below), are indicated in the figure by vertical blue and red lines, respectively.

### Specificity.

The reference standard serum (007sp) was incubated separately with each of the purified PCV-13 PnPSs (3 µg/ml) for 2 h prior to analysis in the assay. Reference standard serum incubated in buffer only was used as a positive control to calculate the percentage of reduction in the IgG concentration in the presence of PnPS competitors. A summary of the specificity data is shown in Table 2. Using homologous PnPS competitors, a ≥80% reduction of the specific antibody concentration relative to the control (serum tested without competitor) was demonstrated for all serotypes. Addition of heterologous PnPS resulted in <20% inhibition for all serotypes, except for the structurally similar 6A/6B and 19A/19F PnPSs, where some level of cross-reactivity was expected and observed. There was an 88% reduction in the concentration of 6A IgG antibodies when 6B PnPS was used as the competitor and a 38% reduction in the concentration of 6B-specific IgG antibodies when 6A PnPS was used as the competitor. Cross-reactivity was shown to be lower between 19F/19A PnPSs, as evidenced by a 33% drop in the level of 19A-specific antibodies with 19F PnPS competitor and a 26% drop in the level of 19F antibodies with 19A PnPS competitor. Similarly, evaluating control sera and selected immunized and nonimmunized adult and infant human sera, acceptable specificity results were observed.

**TABLE 2:** Specificity of 13-plex dLIA

Pneumococcal serotype	% competition of reference standard serum 007sp with PnPs as fluid-phase competitor for serotype^a^:												
	1	3	4	5	6A	6B	7F	9V	14	18C	19A	19F	23F
1	**88**	−1	1	1	1	1	6	8	4	−3	−5	−4	1
3	−4	**100**	−1	−2	−4	−5	−1	2	3	−7	−1	−2	0
4	1	−1	**100**	1	−3	12	6	12	3	−3	−4	11	17
5	8	−2	1	**100**	1	1	8	6	5	−4	−5	−5	−3
6A	−3	0	4	3	**100**	88	6	2	3	−3	−2	0	4
6B	−4	−1	2	0	38	**100**	6	3	3	−3	−3	−3	7
7F	−4	0	1	−1	0	1	**100**	3	4	0	−5	−3	−3
9V	2	−2	−2	−1	−1	0	6	**97**	1	−3	3	−3	0
14	−3	0	2	0	1	1	7	5	**100**	−2	−3	−2	0
18C	−2	−1	0	2	−3	3	3	14	3	**100**	1	0	2
19A	−2	0	1	1	1	2	7	9	5	1	**100**	26	−2
19F	14	−1	3	1	−1	−1	5	15	5	−4	33	**100**	0
23F	−3	0	1	1	0	4	5	1	4	6	−1	0	**100**

a Values shown represent percent reduction of IgG antibody concentration in 007sp reference standard serum in the presence of PnPS competitors at a concentration of 3 µg/ml. Bold data represent PnPs serotypes with homologous inhibition.

The possibility of interference between serotypes in the multiplex assay, especially for serotypes 6A/6B and 19A/19F, was investigated by comparing the calculated specific IgG concentrations in a panel of 33 immune serum samples and three QCS in the 13-plex assay against 13 singleplex assays.

Table 3 shows an analysis of bias between the data generated in singleplex and 13-plex assays. The percentage of mean bias between the 13-plex and singleplex assays fell within the range of 94.6% to 103.9% for all 13 serotypes.

**TABLE 3:** Comparison of mean IgG results between 13-plex and singleplex dLIA

Pneumococcal serotype	% bias of mean IgG concn (13-plex over 1-plex)			
	%RSD	GMR	Lower 90% confidence bound^a^	Upper 90% confidence bound^a^
1	11.0	97.7	94.4	101.0
3	28.3	103.9	95.8	112.8
4	21.9	94.7	88.7	101.0
5	14.6	96.2	92.0	100.5
6A	12.1	95.5	92.2	98.8
6B	14.3	98.9	95.0	103.0
7F	12.6	94.6	91.2	98.2
9V	23.8	96.4	89.9	103.5
14	10.4	94.8	91.2	98.4
18C	13.0	91.1	87.6	94.7
19A	12.1	90.1	86.5	93.8
19F	12.2	99.3	95.4	103.4
23F	15.2	101.5	97.1	106.0

a Data represent results of two one-sided 5% interval tests.

### Validation results and determination of assay range. (i) Accuracy.

For determination of accuracy, a series of mock samples was made from the reference standard serum (007sp) diluted in antibody-depleted human serum (ADHS) and tested eight times. The observed mock sample concentrations were compared to the expected concentrations, based on the known PnPS-specific IgG value, to determine upper and lower IgG concentration limits where the mean bias (expressed as a percentage) of the observed versus expected IgG concentration was between 80% and 125%. Depending on the serotype, the lower and upper limits based on accuracy ranged from 0.004 to 0.032 ng/ml and from 7.250 to 78.600 ng/ml, respectively (Table 4).

**TABLE 4:** Assay range^a^ based on accuracy, dilutional linearity, and precision

Serotype	Well concn^b^ (ng/ml)						Assay range (ng/ml)	
	Accuracy		Dilutional linearity		Precision		Lower limit (ARLL)	Upper limit (ARUL)
	Lower limit	Upper limit	Lower limit	Upper limit	Lower limit	Upper limit		
1	0.010	21.250	**0.015**	24.360	0.005	**18.949**	**0.015**	**18.949**
3	0.004	**7.250**	**0.007**	10.730	0.002	9.462	**0.007**	**7.250**
4	0.012	33.300	**0.020**	**29.150**	0.007	33.208	**0.020**	**29.150**
5	0.013	**18.775**	**0.021**	26.000	0.006	19.978	**0.021**	**18.775**
6A	0.032	78.600	**0.034**	77.371	0.018	**68.207**	**0.034**	**68.207**
6B	0.011	**45.250**	**0.018**	47.070	0.007	46.888	**0.018**	**45.250**
7F	0.009	41.500	**0.010**	**17.321**	0.006	34.277	**0.010**	**17.321**
9V	0.010	32.200	**0.015**	**16.541**	0.007	30.040	**0.015**	**16.541**
14	0.025	**47.488**	**0.071**	56.621	0.011	54.927	**0.071**	**47.488**
18C	0.006	18.250	**0.011**	**16.631**	0.003	18.868	**0.011**	**16.631**
19A	0.017	34.675	**0.028**	40.421	0.008	**36.929**	**0.028**	**36.929**
19F	0.008	36.525	**0.014**	**34.701**	0.004	36.322	**0.014**	**34.701**
23F	0.013	**29.750**	**0.028**	30.740	0.008	37.795	**0.028**	**29.750**

a The lower assay range limit was determined by the most conservative value (highest value for lower limit and lowest value for upper limit) of accuracy, dilution linearity, and precision, highlighted in bold. All of the values represent non-dilution-adjusted “well concentrations.”

b Data represent non-dilution-adjusted concentrations.

### (ii) Dilutional linearity.

Assay ranges for each serotype were also established to define the IgG well concentrations that had acceptable dilutional linearity. Similarly to the accuracy evaluations, the difference between the observed and expected IgG concentration was calculated as the relative bias. IgG concentrations with acceptable bias (80% to 125%) were used to define the assay bounds from bias plots. Depending on the serotype, the lower and upper limits based on the dilutional linearity of 12 human serum samples ranged from 0.007 to 0.071 ng/ml and from 10.730 to 77.371 ng/ml, respectively (Table 4).

### (iii) Precision.

Estimates of assay variability due to day, analyst, and microsphere lot were performed for each of the 44 precision panel samples at dilutions of 1:50, 1:500, and 1:5,000. For each serotype, precision analyses were used to establish the preliminary range of IgG well concentrations that can be measured with acceptable precision (≤25% RSD). Depending on the serotype, the lower and upper limits based on precision ranged from 0.002 to 0.018 ng/ml and from 9.462 to 68.207 ng/ml, respectively (Table 4).

### (iv) Assay range.

The most conservative of the lower and upper IgG concentration limits based on accuracy, dilutional linearity, and precision was used to establish the assay range. The assay range well concentration (Table 4) was adjusted by the use of a sample dilution factor of 50. Table 5 shows the dilution-adjusted assay range in micrograms per milliliter of IgG by serotype, as well as the lower limit of quantitation (LLOQ) and fold range (the ratio of the upper limit of quantitation [ULOQ] and the LLOQ) of the standard curve for each serotype. The LLOQ reflects the lowest measurable IgG concentration in the highest concentration of sample matrix. Samples with values above the ULOQ may be prediluted and retested; therefore, no formal ULOQ was defined for the sample concentration.

**TABLE 5:** Final assay range^a^ and lower limit of quantitation (dilution adjusted)

Serotype	Dilution adjusted assay range (ng/ml)			Fold difference (ARUL/ARLL)
	Lower limit (ARLL)	Upper limit (ARUL)	LLOQ (ng/ml)	
1	0.8	947.5	2	1,184
3	0.4	362.5	4	906
4	1.0	1,457.5	5	1,458
5	1.1	938.8	2	853
6A	1.7	3,410.4	5	2,006
6B	0.9	2,262.5	15	2,514
7F	0.5	866.1	3	1,732
9V	0.8	827.1	13	1,034
14	3.6	2,374.4	5	660
18C	0.6	831.6	2	1,386
19A	1.4	1,733.8	38	1,238
19F	0.7	1,735.1	12	2,479
23F	1.4	1,487.5	9	1,063

a The assay range determined by accuracy, dilution linearity, and precision was adjusted by a sample dilution factor of 50. LLOQ was determined from the precision results from the lowest (1:50) sample dilutions. This approach ensures that the LLOQ reflects the ability of the assay to precisely measure samples in a high-serum matrix.

### (v) Intermediate assay precision.

A variance component analysis (VCA) was performed to evaluate the impact of analyst, assay day, and coated microsphere lot on the overall variability of the assay. Unlike the precision analyses performed to establish the assay range, sample IgG concentrations for intermediate assay precision were derived from those IgG concentrations that fell within the established final assay range. Results are summarized in Table 6. For sample precision, the residual %RSD represents the amount of variability that cannot be ascribed to the analyst, day, or bead lot and reflects assay repeatability. The total %RSD is the estimated intermediate assay precision, which was below 20% RSD for all serotypes.

**TABLE 6:** Intermediate precision

Serotype	*n^a^*	Analyst (%RSD)	Day (%RSD)	Bead lot (%RSD)	Residual (%RSD)	Total (%RSD)
1	702	4.2	4.6	0.4	9.8	11.6
3	702	0.0	4.2	1.8	7.5	8.8
4	704	2.4	4.1	1.7	9.1	10.4
5	702	2.9	4.7	0.3	8.9	10.5
6A	704	2.1	3.6	0.0	9.0	9.9
6B	704	2.9	4.5	1.2	9.0	10.6
7F	702	2.7	4.5	1.7	7.8	9.6
9V	704	2.7	3.5	0.0	9.9	10.9
14	703	4.1	4.8	2.2	8.6	10.9
18C	687	2.6	4.8	0.9	9.3	10.8
19A	686	5.0	3.7	12.2	13.1	19.0
19F	687	2.5	4.6	0.0	10.4	11.6
23F	684	1.5	5.3	2.6	9.6	11.4

a *n*, number of observations, including replicates.

### Stability of PnPS-coated microspheres.

**FIG 2:**
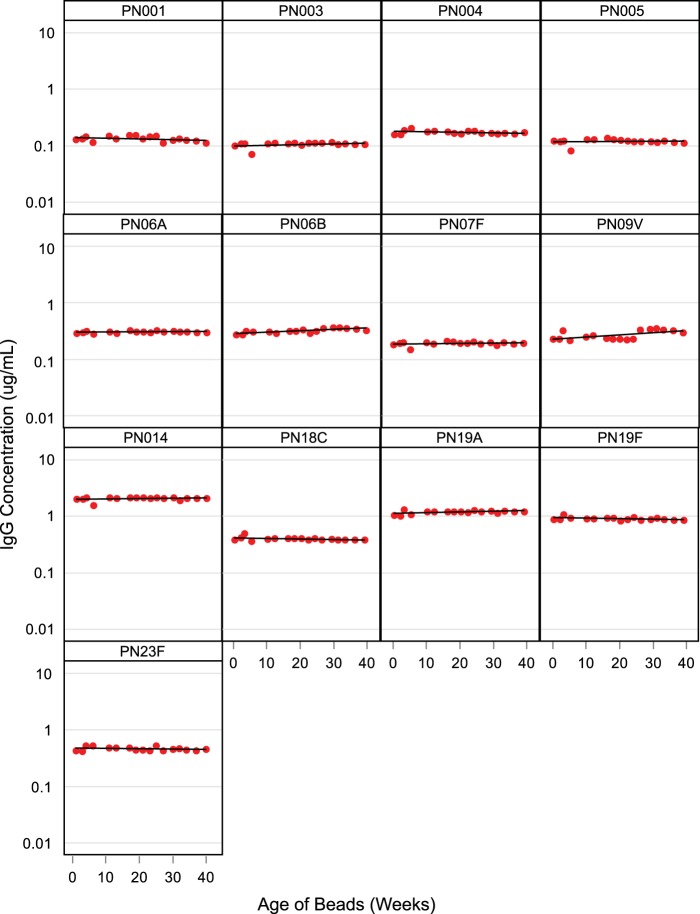
Microsphere stability. Data represent IgG GMC of 44-member serum panel tested on the microspheres over the 40-week period. A proficiency panel of 44 human sera with IgG concentrations ranging from low to high was evaluated on the same microsphere lot over the 40-week period. A single-serum panel was prepared for all 13 serotypes. Each panel (representing one serotype) shows the reported IgG concentrations (*y* axis) of the proficiency panel IgG GMC, as a red dot, over time (*x* axis). A linear trend line is applied to each chart.

The stability of the coated microspheres was monitored post-assay validation by periodic testing of proficiency panel serum samples (*n =* 44) on microspheres stored at 4°C over a 40-week period. The results of the trending analysis are shown in Fig. 2. Each chart shows the geometric mean concentration (GMC) of the serum panel data for a given serotype over the 40-week period. A linear trend line is fitted to the data. The results confirm that PnPS-coated microspheres are stable for 40 weeks.

### Assay robustness and stability.

**FIG 3:**
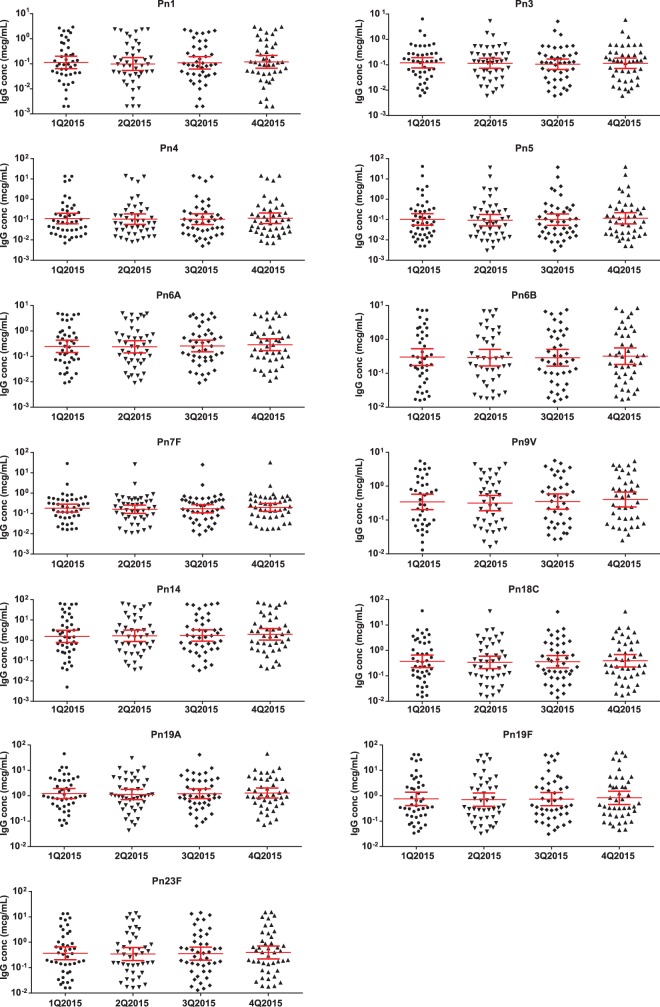
Proficiency panel performance in 13-plex dLIA. A proficiency panel of 44 sera with IgG concentrations ranging from low to high was evaluated post-assay validation quarterly for 1 year. Each panel (representing one serotype) shows the reported IgG concentration (*y* axis) of each of member of proficiency panel sera over time (*x* axis) as a black triangle. GMCs of the serum panels and standard deviation limits are indicated by solid red lines. Assay results are indicated in chronological order from left to right (first quarter 2015 to fourth quarter 2015).

The performance and robustness of the 13-plex dLIA were monitored over time beyond validation through the quality control samples (QCS), which are included on each assay plate and tested in all assay runs, and periodic testing of proficiency panel serum samples (*n =* 44). QCS results were collected from each assay run and showed <20% variability over time (data not shown). Quarterly periodic testing of proficiency panel serum samples was performed for 1 year. Graphs of the proficiency panel data are shown in Fig. 3. Trending analysis showed that the assay had been performing in a consistent manner throughout the 1 year of testing period.

## DISCUSSION

Historically, the standardized WHO ELISA was used to assess PnPS IgG antibody concentrations in sera from subjects enrolled in clinical trials. Increases in the number of serotypes in pneumococcal conjugate vaccines and an increasing number of concomitant vaccine testing requirements in the pediatric population make analysis of antibody responses to the various vaccine components challenging due to the limited volume of serum that can be obtained from infants. Therefore, the availability of methods that can be used with a reduced volume of serum sample is critical. Multiplexed fluorescent microsphere-based assays that have shown to be variable with respect to success in determination of pneumococcal serotype-specific IgG levels have been previously described (9–15, 18–23).

A primary concern of microsphere-based immunoassays is the potential modification of polysaccharide antigen epitopes due to either the conjugation method or the chemical coupling of the polysaccharide to microspheres, which could affect assay sensitivity and specificity. Biagini et al. (9) described the conjugation of periodate-oxidized PnPS to amino groups of adipic acid dihydrazide coupled to EDC/NHS-activated Luminex microspheres. Although those authors claim enhanced sensitivity and dynamic range compared to the ELISA, there were concerns regarding assay specificity for several serotypes, which might have been a result of how the PnPS conjugates were prepared. Pickering et al. (12) and Lal et al. (20) used a cyanuric acid method to conjugate PnPS to PLL. Both groups demonstrated assay specificity and linearity over a wide range of IgG concentrations and good correlation with the WHO ELISA; however, small sample panels were used in the analyses. Attempts to reproduce this method in our laboratory resulted in conjugates with poor reproducibility, and there were safety concerns with the procedure regarding the use of cyanuric chloride.

In the current study, the amination of the PnPS with PLL was performed by conjugation of polysaccharide to PLL by CDAP (1-cyano-4-dimethylaminopyridinium tetrafluoroborate) chemistry (24, 25). The ratio of PnPS to PLL was carefully optimized for each of 13 serotypes to obtain conjugates with adequate PnPS:PLL ratios, which, when used in the dLIA, resulted in specific serum IgG antibody levels that were closely matched to the concentrations measured by ELISA. This optimization was performed using small panels of sera from infants and adults (26–28). The results of those initial bridging experiments were promising and needed to be confirmed by more vigorous experiments using large sets of clinical samples, as was performed in our other study (16).

To confirm that the proposed conjugation of PnPS to PLL is a robust and reproducible process, three lots of PnPS-PLL conjugates corresponding to each of 13 pneumococcal serotypes were prepared and demonstrated outstanding reproducibility. The evaluation of different lots of PnPS-PLL conjugates was performed indirectly, after they were attached to activated carboxylated microspheres, by comparison of PnPS-specific IgG concentrations of proficiency panel sera evaluated in the 13-plex dLIA. Comparison of IgG concentrations of panel sera determined using three lots of PnPS-PLL conjugates demonstrated a GMC (geometric mean concentration) ratio of 80% to 125% between the reference lot and the tested lot for each of the 13 serotypes. Reproducibility and stability for lots of the PnPS-coated microspheres are important factors in the high-throughput process, because having reproducible lots of microspheres with extended stability ensures the consistency of test results from large clinical trials throughout time.

Notable differences between the WHO ELISA and the dLIA developed by Pfizer Inc. are the wider dynamic range of the dLIA platform and the corresponding greater sensitivity and lower LLOQ values across most of 13 serotypes. The nature of the ELISA plate-coating process does not prevent non-PnPS material, which is potentially present in polysaccharide preparations, from adhering to the ELISA plate surface. The chemical coupling process used to coat Luminex microspheres is less prone to attachment of non-PnPS materials, since these are either modified or removed during the conjugate production process, resulting in lower background signal and greater specificity and sensitivity of dLIA (26–28).

The specificity of the 13-plex dLIA demonstrated that the assay detected only PnPS-specific IgG antibodies, which suggests that antigenic epitopes on the PnPS are not adversely affected by the conjugation reaction. Addition of 3 µg/ml of homologous PnPS resulted in over 90% inhibition of signal for tested human sera. Addition of heterologous PnPS resulted in <20% inhibition for all serotypes, except the structurally similar 6A/6B and 19A/19F PnPSs, where cross-reactivity was observed, as expected (29).

Comparison of the IgG concentrations measured by the pneumococcal singleplex and 13-plex assays permitted the determination of interference between the bead sets. This is an important consideration, as there is the possibility of cross-reactivity within multiplexed assays. The geometric mean ratios (GMR) between concentrations of panel of 33 sera determined in the singleplex assays versus the 13-plex assays were 98% (pneumococcal serotype 1 [Pn1]), 104% (Pn3), 95% (Pn4), 96% (Pn5), 96% (Pn6A), 99% (Pn6B), 95% (Pn7F), 96% (Pn9V), 95% (Pn14), 91% (Pn18C), 90% (Pn19A), 99% (Pn19F), and 102% (Pn23F). Overall, these results demonstrated that multiplexing did not appear to alter the concentration of IgG antibodies for the evaluated serotypes.

The current report describes the development and validation of a multiplex Luminex-based immune assay. The 13-plex dLIA uses 007sp reference standard serum (17) to calculate the test serum IgG concentration by log/log-linear regression analysis. Validation experiments confirmed assay accuracy, precision, dilutional linearity, and specificity. The robustness of the assay was exhibited throughout a year using a panel of the sera and confirmed reliable assay performance. The suitability of this new method against the WHO ELISA was successfully verified (16).

In conclusion, a 13-plex immunoassay for simultaneous measurement of IgG antibodies against 13 PnPSs in human serum samples was developed and validated. This immunoassay provides an accurate, sensitive, efficient, and cost-effective high-throughput analysis of serum samples and is a suitable replacement for the WHO standardized ELISA. The PnPS dLIA provides a platform that is suitable to use and flexible for future additions of new antigens with an expansion of serotypes.

## MATERIALS AND METHODS

The human reference standard serum, 007sp (17), was provided by the Center for Biological Evaluation and Review, U.S. Food and Drug Administration. Panels of serum samples and quality control samples (QCS) were established using human serum samples from adult sera immunized with Pneumovax II (23vPPV; Merck) or Prevnar 13 (13vPnC; Pfizer).

### Coupling of PnPS-PLL conjugates to the magnetic carboxylated microspheres.

Capsular PnPS serotypes 1, 3, 4, 5, 6A, 6B, 9V, 14, 18C, 19A, 19F, and 23F (Pfizer Vaccine Research and Development, Pearl River, NY) are aminated by conjugation to poly-l-lysine (hydrobromide) (PLL; Sigma) using 1-cyano-4-dimethylaminopyridinium tetrafluoroborate (CDAP; Sigma) conjugation chemistry (24, 25). The PnPS are activated with CDAP as follows. CDAP (J.T. Baker; catalog no. 9017-02) is added to the PnPS solution in water. After addition of triethylamine (TEA; Sigma-Aldrich), bicarbonate buffer (pH 8.9) is added. Poly-l-lysine (PLL) is added to the activated PnPS, and the reaction mixture is incubated overnight (16 to 24 h) at 2 to 8°C with slow mixing. After the incubation, the reaction mixtures are clarified by centrifugation to remove any insoluble material. The resulting PnPS-PLL conjugates are purified by size exclusion chromatography. Fractions are analyzed for the presence of the PnPS and PLL using colorimetric assays.

PnPS-PLL conjugates are coupled to the spectrally unique magnetic carboxylated microspheres (Luminex, TX, USA) using a modification of a coupling described by Luminex (catalog no. 40-50016) and a method published by Pickering et al. (12). Prior to coupling, the carboxyl groups on the surface of the polystyrene beads are activated with a carbodiimide derivative, 1-ethyl-3-(3-dimethylaminopropyl) carbodiimide hydrochloride (EDC; Pierce). The unstable intermediate is stabilized in aqueous solutions using sulfo-N-hydroxysulfosuccinimide (sNHS; Pierce). These intermediates react with primary amines of proteins (poly-l-lysine from the PnPs-PLL conjugate) to form amide bonds. Briefly, microspheres are suspended in HEPES buffer and 10 µg of EDC and sNHS is added to the microsphere suspension and incubated on a nutating mixer. PnPS-PLL conjugate is added to the activated microspheres. The concentration of PnPS-PLL conjugate added to the reaction mixture was optimized for each of the 13 PnPS-PLL conjugates. The microspheres and PnPS-PLL conjugates are incubated for 120 ± 30 min at 18 to 25°C on a Rotamix rotator (Rotamix; ATR Inc.) at 25 rpm. After unbound antigen is removed by washing (phosphate-buffered saline [PBS; pH 7.4] containing 1% bovine serum albumin [BSA] and 0.05% sodium azide), the microspheres are stored away from direct light in PBS buffer (pH 7.4) containing 1% BSA and 0.05% sodium azide. A summary of the microsphere coupling conditions is listed in Table 7.

**TABLE 7:** Summary of microsphere coupling conditions

Parameter	Value
EDC/sNHS concn (µg/ml)	5,000
Microsphere activating time (min)	20
Coupling buffer	50 mM HEPES
PnPS-PLL coupling concn (µg/ml)	0.5–1 µg/ml (based on serotype)
Coupling incubation time (min)	120
Bead concn in coupling reaction	1.25 × 10^7^/0.5 ml
Storage buffer	1% BSA–DPBS

### 13-plex direct Luminex immunoassay procedure.

The 13-plex dLIA was designed on the basis of Luminex MagPlex xMAP technology. All test serum samples, reference standard serum 007sp, and QCS are diluted in phosphate-buffered saline (PBS buffer; pH 7.2) containing two pneumococcal cell wall polysaccharide (CWPS) absorbents (CWPS1 and CWPS2; Pfizer) (5 and 1 µg/ml, respectively) termed double-absorbent buffer (30–32) and incubated for 1 to 2 h at room temperature (18 to 25°C). The assay has an 11-point, 2.5-fold dilution, standard curve that uses the international reference standard 007sp, which has published weight-based antibody concentrations (expressed in micrograms per milliliter) (17) and three control sera on each plate. Test samples are tested at three 10-fold dilutions with a 50-fold starting dilution. Samples that fall above the assay range can be retested at higher dilutions. A 50-μl volume of antigen-coated microspheres (pooled mixture of ~2,500 microspheres per serotype) is dispensed into a 96-well flat-bottom opaque white assay plate (Costar; Corning Inc., Corning, NY). All serum dilutions are added to the assay plate (50 µl/well) and incubated for 90 (±15) min at 18 to 25°C. After washing with PBS buffer (pH 7.4) containing 0.02% NaN_3_ and 0.05% Tween 20 to remove nonbound components, a purified R-phycoerythrin-labeled goat anti-human IgG secondary antibody (Jackson ImmunoResearch Laboratories, Inc., West Grove, PA) is added to the microsphere mixture followed by incubation for 90 (±15) min at 18 to 25°C. After the final wash, 100 µl of PBS is added to each well and the plates are read on a Luminex reader. Raw data are expressed as median fluorescent intensities (MFIs), and IgG antibody concentrations are calculated from interpolation of sample MFI values to the reference standard curve that is fitted by a log/log-linear regression model.

### Assay specificity.

The specificity of the 13-plex dLIA is assessed by competitive inhibition experiments. Briefly, homologous, heterologous, or nonrelated polysaccharide competitors are added to serum samples that have been diluted in the double-absorbent antibody dilution buffer. Following incubation, the absorbed serum samples are transferred to the assay plates and tested in the 13-plex dLIA. Specificity is expressed as the percentage of reduction in serotype-specific IgG concentration relative to no competitor, as follows: specificity (%) = [(PnPS-specific serum IgG concentration – PnPS-serum IgG concentration absorbed with competitor)/PnPS-specific serum IgG concentration] × 100.

The assay was determined to be specific for the target PnPS antigen when ≥80% homologous inhibition and ≤20% heterologous/unrelated inhibition were observed.

### Assay validation.

Assay validation consisted of a series of experiments to address accuracy, dilutional linearity, and precision (33, 34). For the assessment of accuracy, mock samples were prepared by diluting the 007sp international reference standard in matrix (antibody-depleted human serum [ADHS]) to create a series of 11 independent samples. The 11 accuracy samples were then diluted 50-fold, 500-fold, and 5,000-fold to generate a panel of 33 dilutions that were tested on a single assay plate. Mock samples were prepared so that the expected titers spanned the preliminary assay range determined during assay prevalidation. Accuracy experiments were performed by a single operator on eight independent plates containing the reference standard, control sera, and 33 accuracy mock samples. The observed sample concentrations were compared to the expected concentrations to determine the accuracy of each mock sample, and the percentage of bias (100 × observed concentration/expected concentration) was determined. Accuracy was considered acceptable if the bias was between 80% and 125%.

Dilutional linearity of the 13-plex dLIA was established with a panel of 12 individual adult or infant postimmune serum samples that spanned a range (high, medium, and low) of PnPS-specific IgG antibody concentrations for each of 13 serotypes. These samples were tested eight times across a series of 11 dilutions. The results were incorporated into plots of relative bias versus expected well concentration (not adjusted by serum dilution). A smoothing spline curve was fitted to the relative bias data. The lower and upper limits, based on dilutional linearity, were defined as the ranges of expected antibody titers that had acceptable relative bias. This range was determined by the intersection of the mean relative bias, as defined by the smoothing spline, and the acceptance limits of 80% to 125%.

Precision data describe the closeness of measurements for a sample tested multiple times. Precision is a measure of assay variability that includes both repeatability and intermediate precision. Repeatability measures the assay variability over a wide range of antibody concentrations, whereas intermediate precision measures the variability within the defined assay range due to relevant sources of variability (e.g., different analysts, times, and coated microsphere lots).

Precision was established by evaluating 44 individual human serum samples that had been obtained from immunized adults and infants. Precision experiments were run over multiple days, with multiple operators using independently prepared lots of coated microspheres. The total variability for each sample, expressed as percent relative standard deviation (%RSD), was plotted against the observed IgG non-dilution-adjusted IgG concentration, and a LOESS (local regression) curve was fitted to each plot (35). The lower and upper IgG concentration limits, based on precision, were defined as the lowest and highest IgG concentrations at which the LOESS curve remained equal to or below the acceptable variability of 25%.

The assay range for each serotype was based on the most conservative values from the lower and upper IgG concentration limits with acceptable accuracy (bias ratio between 80% and 125%), dilutional linearity (bias ratio between 80% and 125%), and precision (%RSD less than or equal to 25%). To determine the lower level of quantitation (LLOQ), the precision results from the least diluted samples were used to account for the highest matrix concentration.

The total %RSD (intermediate precision) was calculated by combining the individual components of variance from a variance component analysis (VCA). The VCA estimates variability due to analyst, day, and coated microsphere lot.
